# Supplementary material for: The antimicrobial potential of traditional remedies of Indigenous peoples from Canada against MRSA planktonic and biofilm bacteria in wound infection mimetic conditions
Source: Microbiol Spectr. 2024 Nov 12;12(12):e02341-24. doi: 10.1128/spectrum.02341-24 (PMC11619235; doi:10.1128/spectrum.02341-24)
Supplement: Supplemental material — Figures S1 to S3; Tables S1 and S2. [file spectrum.02341-24-s0001.docx]

**Supplementary Figures and Tables**

**The antimicrobial potential of traditional remedies of Indigenous Peoples from Canada against MRSA planktonic and biofilm bacteria in wound-infection mimetic conditions**

**Colin D. Rieger^1^, Ahmed M. Soliman^1,2^, Kateryna Kaplia^3,4^, Nilrup Ghosh^3,5^, Alexa Cervantes Lopez^3,6^, Surya Arcot Venkatesan^3,7^, Abraham Gildaro Guevara Flores^3,8^, Matheus Antônio Filiol Belin^3, 9^, Florence Allen^10 †^, Margaret Reynolds^11^, Betty McKenna^12^, Harold Lavallee^13 ††^, Archie Weenie^14^, Thomas Favel^15†††^, Fidji Gendron^3^, Vincent E. Ziffle^3^ and Omar M. El-Halfawy^1,16*^**

^1^ Department of Chemistry and Biochemistry, Faculty of Science, University of Regina, Regina, SK, S4S 0A2, Canada

^2^ Department of Microbiology and Immunology, Faculty of Pharmacy, Kafr-Elsheikh University, Kafr El-Sheikh 33516, Egypt

^3^ Department of Indigenous Knowledge and Science, Faculty of Science, First Nations University of Canada, Regina, SK, S4S 7K2, Canada

^4^ Kingston University, Faculty of Health, Science, Social Care and Education, KT1 2EE, UK

^5^ IISER Kolkata, West Bengal 741246, India

^6^ Instituto Tecnológico y de Estudios Superiores de Monterrey, 64849 Monterrey, N.L, Mexico

^7^ Engineering and Technology, Rajalakshmi Engineering College, Kanchipuram, Tamil Nadu, 602105, India

^8^ Autonomous University of Nuevo Leon (Medicine College), Department of Chemistry, 66455 San Nicolás de los Garza, N.L., Mexico

^9^ Universidade Estadual Paulista (UNESP), Campus de Botucatu. Faculdade de Medicina (FMB), 18618-970, Brazil

^10^ Elder from Peter Ballantyne Cree Nation in Saskatchewan, Treaty 6 Territory, Canada

^11^ Elder from English River First Nation in Saskatchewan, Treaty 10 Territory, Canada

^12^ Elder from Shoal River Band in Manitoba, Treaty 4 Territory, Canada

^13^ Elder from Piapot First Nation in Saskatchewan, Treaty 4 Territory, Canada

^14^ Elder from Sweetgrass First Nation in Saskatchewan, Treaty 6 Territory, Canada

^15^ Elder from Kawacatoose First Nation in Saskatchewan, Treaty 4 Territory, Canada

^16^ Department of Microbiology and Immunology, Faculty of Pharmacy, Alexandria University, Alexandria, 21521, Egypt

^†^Deceased on 7 January 2023

^††^Deceased on 3 July 2022

^†††^Deceased on 2 September 2024

**^*^ Correspondence: omar.el-halfawy@uregina.ca (O.M.E.)**

**Running title: The antimicrobial potential of Indigenous remedies against MRSA**

**
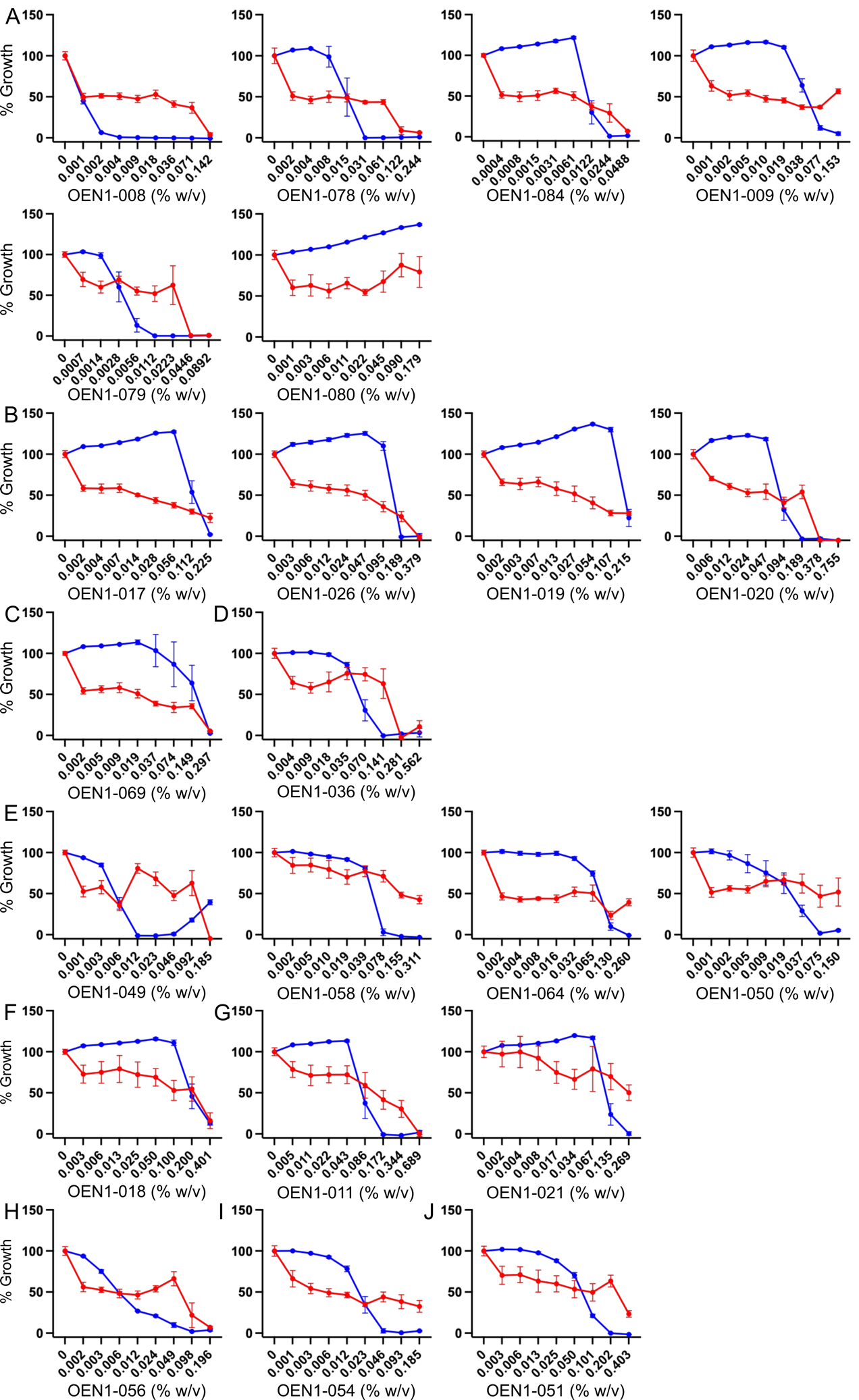
**

**Figure S1. Dose-response assays of Indigenous remedies’ extracts** from A) bergamot (horsemint) OEN1-008, -078, -084, -009, -079, and -080; B) gumweed OEN1-017, -026, -019, and -020; C) spreading dogbane OEN1-069; D) labrador tea OEN1-036; E) dock OEN1-049, -058, -064, and -050; F) gaillardia OEN1-018; G) pasture sage OEN1-011 and -021; H) rose hip OEN1-056; I) wild raspberry OEN1-054; J) dandelion OEN1-051. Dose-response curves in SWF (red) and MHB (blue). N=6 from 3 independent experiments shown as mean of the percent growth ± SEM.


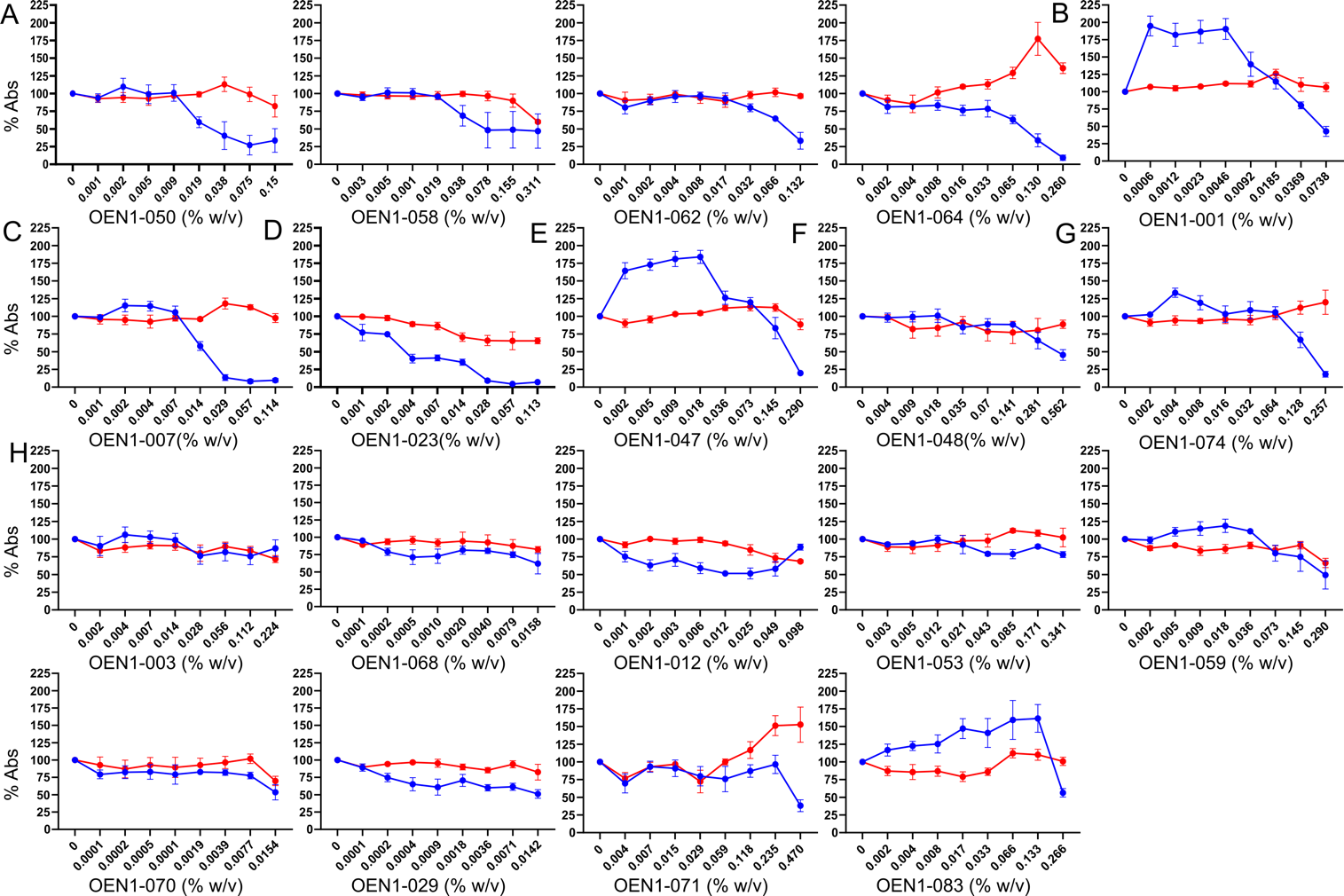


**Figure S2. Dose-response assays of** **Indigenous remedies’ extracts for biofilm prevention activity against MRSA.** A) Dock OEN1-050, -058, -062, and -064; B) prairie coneflower OEN1-001; C) Gaillardia OEN1-007; D) wild red raspberry OEN1-023; E) goldenrod OEN1-047; F) rabbit root OEN1-048 G) rose hip OEN1-074; H) represent extracts without biofilm prevention activity in TSB or SWF. Y-axis represents percent A_590_ of USA300 biofilm treated with the corresponding extract relative to an untreated control. Dose-response curves are in SWF (red) and TSB (blue). The results were shown as mean percent absorbance ± SEM.


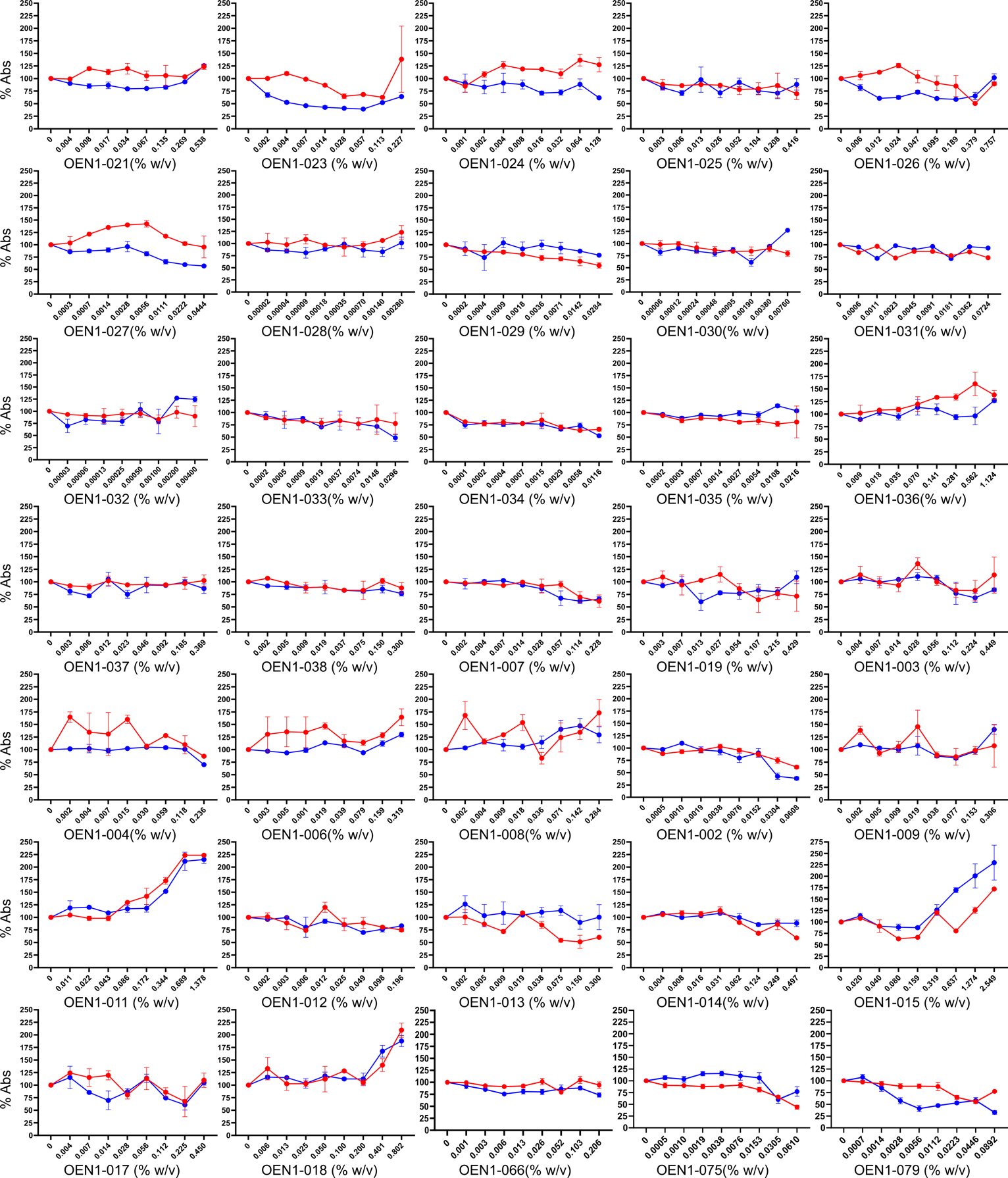


**Figure S3. Dose-response assays of** **Indigenous remedies’ extracts for biofilm eradication activity against MRSA.** The figure represents extracts without notable activity either in TSB or in SWF. Y-axis represents percent A_590_ of USA300 pre-formed biofilm treated with the corresponding extract relative to an untreated control. Dose-response curves are in SWF (red) and TSB (blue). The results were shown as mean percent absorbance ± SEM.

**Table S1.** MIC values (µg/mL) of control antibiotics tested against SH1000 and CRP strains cultured in SWF or MHB

| **Strain tested** | **Control antibiotic tested** | **CRP strain** | | **SH1000** | | **Resistant to antibiotic class** |
| --- | --- | --- | --- | --- | --- | --- |
|  |  | **MHB** | **SWF** | **MHB** | **SWF** |  |
| AJUL1 | chloramphenicol | >64 | >64 | 8 | 8 | phenicols |
| AJUL2 | kanamycin | >16 | >16 | 0.5 | 0.5 | aminoglycosides |
| AJUL5 | kanamycin | >16 | >16 | 0.5 | 0.5 | aminoglycosides |
| AJUL6 | streptomycin | 512 | 32 | 16 | 1 | aminoglycosides |
| AJUL7 | retapamulin | >0.5 | >0.5 | 0.125 | 0.0625 | Phenicols, linxosamides,  oxazolidinones, pleuromutilins, steptogramins (A) |
| AJUL8 | erythromycin | >2 | >2 | 0.5 | 0.125 | marolides, lincosamides,  streptogramins (A) |
| AJUL10 | erythromycin | >2 | >2 | 1 | 0.125 | marolides, lincosamides,  streptogramins (A) |
| AJUL11 | fusidic acid | 32 | >256 | 0.5 | 16 | fusidic acid |
| AJUL12 | mupirocin | >32 | >32 | 1 | 4 | mupirocin |
| AJUL14 | tetracycline | 16 | >64 | 2 | 8 | tetracyclines |
| AJUL15 | tetracycline | 8 | >64 | 2 | 8 | tetracyclines |
| AJUL16 | retapamulin | >0.5 | >0.5 | 0.125 | 0.0313 | pleuromutilins |
| AJUL18 | bacitracin | >512 | >512 | 32 | 16 | bacitracin |
| AJUL19 | ampicillin | 8 | 8 | 0.5 | 0.5 | β-lactams  (penicillinase-susceptible) |
| AJUL20 | oxacillin | >8 | 4 | 1 | 0.5 | β-lactams  (penicillinase-stable) |
| AJUL21 | fosfomycin | >512 | >512 | 128 | 32 | fosfomycin |
| AJUL22 | daptomycin | 16 | 4 | 4 | 1 | daptomycin |
| AJUL23 | rifampicin | >0.5 | >0.5 | 0.0078 | 0.0625 | rifamycins |
| AJUL24 | trimethoprim | >256 | >256 | 4 | >256 | diaminopyrimidines |
| AJUL25 | sulfamethoxzole | >2048 | >2048 | >2048 | >2048 | sulphonamides |
| AJUL26 | ciprofloxacin | 64 | 16 | 0.5 | 0.5 | fluoroquinolones |
| AJUL27 | novobiocin | >64 | >64 | 16 | 0.25 | aminocoumarins |
| AJUL28 | triclosan | 2 | 128 | 0.125 | 2 | triclosan |

**Table S2.** MIC values (% w/v) of Indigenous remedies’ extracts tested against SH1000 cultured in SWF or MHB

| Extract code | MIC against SH1000 (% w/v) | |
| --- | --- | --- |
|  | SWF | MHB |
| Extracts that inhibited USA300 with higher potency in SWF than MHB | | |
| OEN1-022 | 0.415 | 0.415 |
| OEN1-067 | 0.225 | >0.225 |
| OEN1-053 | 0.341 | 0.341 |
| OEN1-063 | 0.231 | > 0.231 |
| OEN1-070 | > 0.015 | > 0.015 |
| Extracts that inhibited USA300 with relatively comparable potency in SWF and MHB | | |
| OEN1-058 | > 0.311 | 0.155 |
| OEN1-021 | > 0.269 | > 0.269 |
| OEN1-008 | 0.142 | 0.142 |
| OEN1-084 | 0.024 | 0.012 |
| OEN1-078 | 0.122 | 0.061 |
| OEN1-079 | 0.045 | 0.022 |
| OEN1-069 | 0.149 | 0.074 |
| OEN1-049 | 0.092 | 0.023 |
| OEN1-064 | 0.260 | 0.260 |
| OEN1-050 | > 0.150 | 0.075 |
| OEN1-011 | 0.344 | 0.344 |
| OEN1-036 | 0.141 | 0.141 |
